# Effects of Melanized Bacteria and Soluble Melanin on the Intestinal Homeostasis and Microbiome In Vivo

**DOI:** 10.3390/toxics11010013

**Published:** 2022-12-23

**Authors:** Yong-guo Zhang, Mackenzie E. Malo, Tanya Tschirhart, Yinglin Xia, Zheng Wang, Ekaterina Dadachova, Jun Sun

**Affiliations:** 1 Division of Gastroenterology and Hepatology, Department of Medicine, University of Illinois Chicago, Chicago, IL 60612, USA; 2 College of Pharmacy and Nutrition, University of Saskatchewan, Saskatoon, SK S7N 5E5, Canada; 3 Center for Bio/Molecular Science and Engineering, U.S. Naval Research Laboratory, Washington, DC 20375, USA; 4 Department of Microbiology and Immunology, University of Illinois Chicago, Chicago, IL 60612, USA; 5 University of Illinois Chicago (UIC) Cancer Center, University of Illinois Chicago, Chicago, IL 60612, USA; 6 Jesse Brown VA Medical Center, Chicago, IL 60612, USA

**Keywords:** *E. coli* Nissle, intestine, melanin, melanized bacteria, microbiome, probiotics

## Abstract

Radiation damage is associated with inflammation and immunity in the intestinal mucosa, including gut microbiota. Melanin has a unique capacity to coordinate a biological reaction in response to environmental stimuli, such as radiation exposure. Thus, melanin and melanized microbes have potential to be used for mitigation of injury induced by radiation. The purpose of the current study is to examine the safety of these agents for future targeting gut microbiome to prevent radiation-induced injury. We administered mice with soluble allomelanin and observed its effect on the intestinal physiology and body weight. We then established a melanized bacterial strain in probiotic *E. coli* Nissle. We measured the body weight of the mice treated with melanized *E. coli* Nissle. We showed the enhanced bacterial abundance and colonization of the melanized bacteria *E. coli* Nissle in the intestine. Melanized *E. coli* Nissle colonized the colon in less than 3 h and showed consistent colonization over 24 h post one oral gavage. We did not find significant changes of bodyweight in the mice treated with melanized bacteria. We did not observe any inflammation in the intestine. These results demonstrate the safety of soluble melanin and melanin-producing bacteria and will support the future studies to treat radiation-induced injuries and restore dysbiosis.

## 1. Introduction

Melanin is a diverse group of pigments identified in all biological kingdoms [1]. It is a complex biopolymer generated from a variety of precursor materials, using different biosynthetic pathways. Melanin has advantageous functions in extreme conditions, including radiation exposure [1]. Melanin can be divided into 5 categories: eumelanin, neuromelanin, pheomelanin, pyomelanin, and allomelanin [2]. The first 3 are predominantly found in animals, whereas the pyomelanin and allomelanin exist predominantly in bacteria, fungi, and plants [3,4]. Melanized fungi have survived in extreme locations such as the International Space Station, the Chernobyl atomic energy station, and Antarctic deserts, which are locations with elevated radiation levels, and under these circumstances melanized fungi outnumber their non-melanized counterparts, suggesting a selective advantage for melanin [5,6,7,8,9]. First, this selective advantage works through melanin’s ability to provide physical shielding by improving structural integrity and enabling compton scattering [10,11,12,13]. Second, melanin plays a role in chemical shielding as an antioxidant and free-radical scavenger [14,15,16]. Finally, melanin imparts advantage through its capacity to coordinate a biological reaction in response to environmental stimuli [17,18,19,20]. Thus, soluble melanin and melanized microbes have potential to be used for mitigation of injury induced by radiation.

The gut microbiome represents the collective genomes of a complex and necessary ecosystem, composed of trillions of living microorganisms in the intestine. This so called “microbiome organ” weighs over 1 kg, equivalent to the weight of the human heart or liver, whereas it has no distinct structure, organized system of microbiome cells is more akin to immune system. The functions of microbiome, an invisible organ, include (i) synthesizing nutritional factors; (ii) producing anti-microbial products; (iii) developing a systemic and intestinal immune system; (iv) supporting epithelial renewal and maintaining barrier functions; and (v) detoxifying xenobiotics and affecting the host metabotypes [21,22,23,24]. Thus, microbiome is critical in health and disease [25,26,27]. There is emerging evidence implicating the gut microbiome in the pathogenesis of radiation-induced injuries and dysbiosis increases the intestinal susceptibility to injuries [25,28]. Radiation damage is associated with inflammation and immunity in the gut mucosa, including microbiome [25,28]. Therefore, targeting microbiome is anticipated to prevent radiation-induced injury.

In the current study, we evaluated the effects of soluble melanin and melanized bacteria on the intestinal homeostasis and microbiome *in vivo* with the goal of evaluating the safety of these agents. We administered mice with soluble allomelanin and observed its effect on the intestinal physiology and bodyweight. We then established melanized bacteria in probiotic *E. coli* Nissle. We measured the body weight of the treated mice and did not find significant changes of body weight in the mice treated with melanized bacteria. We did not observe any inflammation in the intestine. We showed the enhanced bacterial abundance and colonization of the melanized bacteria in the intestine. Our research will help the future studies to treat radiation-induced injuries and restore dysbiosis.

## 2. Materials and Methods

### 2.1. Allomelanin Administration in Mice

Animal studies were approved by the Animal Research Ethics Board of the University of Saskatchewan (animal use protocol #20190028, approved on 19 April 2019). Male and female CD1 mice aged 7 weeks were purchased (Charles River Laboratories, Kingston, ON, Canada) and maintained in sterile housing at the University of Saskatchewan.

At 17 weeks old mice were fasted overnight but allowed water ad libitum. Soluble allomelanin was purchased from Karpathia Trust Fund Inc., Cleveland, OH, USA. This allomelanin was derived from *Inonotus obliquus* (common name Chaga mushroom). The fungal melanin was subsequently modified by the manufacturer to increase the number of paramagnetic centers while preserving its water solubility. Melanin solution was a true solution which preserved its homogeneity even after prolonged ultracentrifugation. Fasted mice were administered a 12.5 mg/mL sterile solution of soluble allomelanin in water as a single oral bolus of 0.2 mL, and control mice received a single 0.2 mL oral bolus of sterile water. Immediately following oral gavage mice were placed in sterile empty cages, and the first 2 fecal pellets were collected. Fecal pellets were collected with sterile pipet tips, transferred into sterile microcentrifuge tubes, and then frozen at −80 °C. Mice were then returned to standard sterile housing with their cage mates with access to chow and water ad libitum. Sterile fecal pellet collection was repeated at 2/12/24 h post administration of allomelanin as described above.

### 2.2. Establish Melanized Bacteria in Probiotic E. coli Nissle

We chose to probiotic *E. coli* Nissle to express melanin because this strain has been used in the clinical to treat the intestinal disorders [29]. The plasmid pJV-Tyr1 was constructed as reported in the previous publication [30]. It contains the synthesized tyrosinase gene from *Bacillus megaterium Tyr1* under the control of the inducible promoter *Ptac*. This plasmid was transformed into the probiotic *E. coli* Nissle strain prepared with the room temperature electroporation protocol. *E. coli* Nissle (pJV-Tyr1) was grown overnight from a glycerol stock in LB medium with 20 µg/mL chloramphenicol at 37 °C and 250 rpm. A 0.5 mL aliquot of the overnight culture of *E. coli* Nissle (pJV-Tyr1) was transferred into 50 mL LB medium supplemented with 10 mg/mL chloramphenicol and incubated at 200 rpm at 37 °C for 3 h. Tyrosinase production was induced by the addition of 200 μM isopropyl-β-d-1-thiogalactopyranoside (IPTG) for three more hours. The melanized bacterial cells were prepared by adding 50 mg/mL CuSO4 and 0.4 mg/mL L-tyrosine into the culture and shaken at 37 °C for 24 h (non-melanized bacterial cells were prepared by omitting L-tyrosine in the same culture). The melanized bacterial cells were harvested by centrifugation and resuspended in PBS buffer.

### 2.3. Bacterial Treatment In Vivo

CD-1 mice (male and female, 12 weeks) were gavaged with probiotic *E. coli* Nissle or melanized *E. coli* Nissle (1 × 10^7^ cfu), which was engineered to heterologously express tyrosinase gene (*tyr1*). Fecal samples were collected at the 0, 3, 12, and 24 h post treatment. The animal work was approved by the UIC Office of Animal Care (ACC 21-120).

### 2.4. Histology

Colon and ileum were fixed in 10% neutral buffered formaldehyde overnight, and then held in 70% ethanol until processing. Tissues were paraffinized and sectioned at 4 μm by microtome. The slides were stained with hematoxylin and eosin.

### 2.5. E. Coli Nissle Culture in Feces

Feces (about 20 mg) collected from each mouse were put into 1.5 mL tubes with 1 mL of sterile phosphate-buffered saline (PBS), and then vortexed adequately. Each sample in 5, 50, 500 and 5000 dilution with LB medium were plated (100 μL) on LB Agar plates with 10 µg/mL chloramphenicol, and grown overnight at 37 °C. Colony-forming units were quantified.

### 2.6. Real-Time PCR Measurement of Bacterial DNA

From mouse feces, DNA was extracted using EZNA Stool DNA Kit (Omega Bio-tek, Inc. D4015-01, Norcross, GA 30071, USA). The quantitative real-time PCR was conducted using the CFX96 Real-time PCR detection system and iTaqTM Universal SYBR green supermix (Bio-Rad Laboratories, 1725121, Hercules, CA, USA). All expression levels were normalized to universal bacteria levels of the same sample. All real-time PCR reactions were performed in triplicate. Primer sequences were designed using Primer-BLAST or obtained from Primer Bank (Table 1).

### 2.7. Statistical Analysis

All data were expressed as the mean ± SD. All statistical tests were 2-sided. All *p*-values < 0.05 were considered statistically significant. Based on data distributions, the differences between samples were analyzed using Welch’s *t*-test for two groups and one-way ANOVA for more than two groups as appropriate, respectively. The differences between groups over post treatment were analyzed using two-way ANOVA. Adjusting for multiple comparisons to correct *p*-values was performed by Tukey method in both one-way and two-way ANOVA tests. Statistical analyses were performed using GraphPad Prism 8 (GraphPad, Inc., San Diego, CA, USA).

## 3. Results

### 3.1. Soluble Melanin Treatment In Vivo

**Figure 1:**
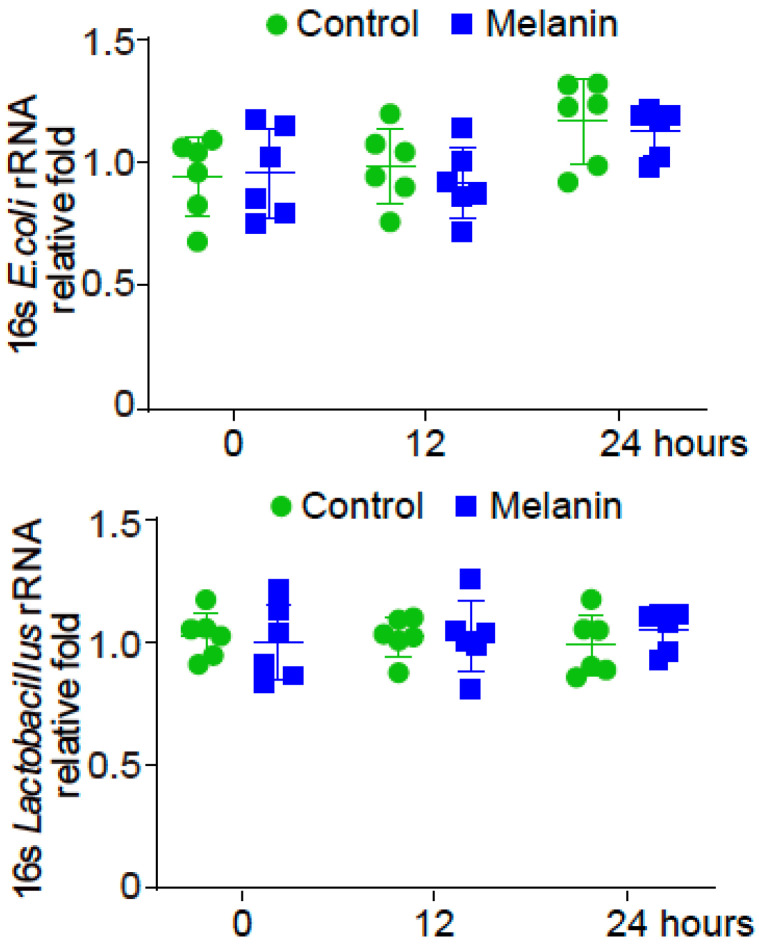
Gut microbes in the soluble melanin-treated CD-1 mice. The compositions of *Escherichia coli* and *Lactobacillus* did not change post 12 and 24 h after CD-1 mice treated with melanin (2.5 mg/mouse), compared to the untreated mice group. Data are expressed as mean ± SD. N = 6, two-way ANOVA test and the *p*-values were adjusted with Tukey method for multiple comparisons.

We treated the CD-1 mice with soluble melanin (2.5 mg/mouse). We did not observe any body weight change in these mice. We then examined the intestinal microbiome. The compositions of *Escherichia coli* and *Lactobacillus* did not change 12 and 24 h post treatment, compared to the untreated mice group (Figure 1).

### 3.2. Microbial Changes in Melanized Bacteria Treated Mice

**Figure 2:**
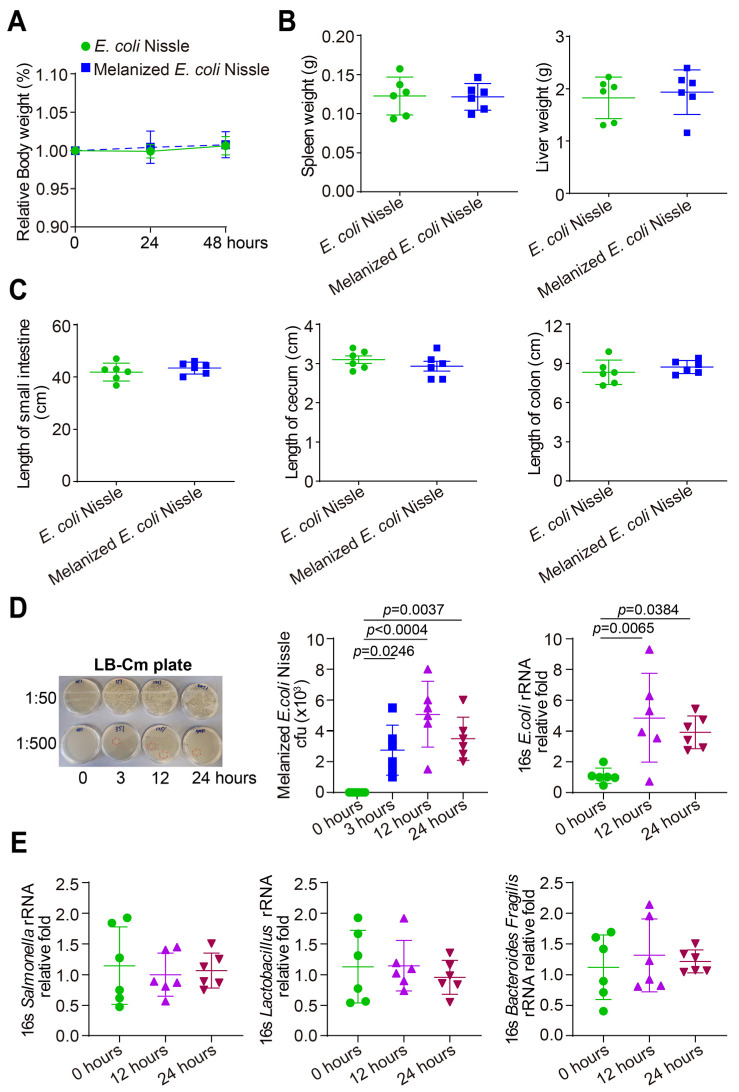
CD-1 mice were one-gavaged with probiotic *E. coli* Nissle or melanized *E. coli* Nissle. (**A**) Relative body weight did not change in CD-1 mice gavaged with probiotic *E. coli* Nissle or melanized *E. coli* Nissle. Data are expressed as mean ± SD. N = 6, two-way ANOVA test and the *p*-values were adjusted with Tukey method for multiple comparisons. (**B**) The spleen and liver weight did not change significantly in CD-1 mice gavaged with probiotic *E. coli* Nissle or melanized *E. coli* Nissle post 48 h. Data are expressed as mean ± SD. N = 6, Welch’s *t* test. (**C**) The length of small intestine, cecum and colon did not change in CD-1 mice gavaged with probiotic *E. coli* Nissle or melanized *E. coli* Nissle post 48 h. Data are expressed as mean ± SD. N = 6, Welch’s *t* test. (**D**) The melanized *E. coli* Nissle in feces post one-gavage was tested by culture and PCR. The melanized *E. coli* Nissle (LB cm agar plates, 10 µg/mL) was cultured using one-gavaged mice fecal samples. The melanized bacteria could be detected at 3, 12, and 24 h post treatment. The number of melanized bacteria increased at 3, 12, and 24 h post treatment compared to the before treatment in CFU count. The *E. coli* 16S rRNA RT-PCR amplification indicates the increased *E. coli and* melanized *E. coli* Nissle in fecal samples. Data are expressed as mean ± SD, N = 6, one-way ANOVA test and the *p*-values were adjusted with Tukey method for multiple comparisons. All *p* values are shown in this figure. (**E**) The compositions of *Salmonella*, *Lactobacillus* and *Bacteroides Fragilis* did not change in the melanized bacteria-treated mice. Data are expressed as mean ± SD. N = 6, one-way ANOVA test and the *p*-values were adjusted with Tukey method for multiple comparisons.

In order to get the consistent melanin in the intestine, we started to establish melanized bacteria in probiotic *E. coli* Nissle. We chose to probiotic *E. coli* Nissle to express melanin because this strain has been used in the clinical to treat the intestinal disorders [29]. CD-1 mice (male and female) were gavaged with probiotic *E. coli* Nissle or melanized *E. coli* Nissle, which was engineered to heterologously express tyrosinase gene (*tyr1*). As shown in Figure 2A, body weight did not change in CD-1 mice gavaged with probiotic *E. coli* Nissle or melanized *E. coli* Nissle. The spleen and liver weight did not change significantly in CD-1 mice gavaged with probiotic *E. coli* Nissle or melanized *E. coli* Nissle post 48 h (Figure 2B). The length of small intestine, cecum and colon did not change in CD-1 mice gavaged with probiotic *E. coli* Nissle or melanized *E. coli* Nissle post 48 h (Figure 2C). Fecal samples were collected at the 0, 3, 12, and 24 h post treatment. The melanized *E. coli* Nissle in feces was tested by culture and PCR. As shown in Figure 2D, the melanized bacteria could be detected 3, 12, and 24 h post treatment, indicating its ability to colonize in the intestine in less than 3 h and continued colonization 24 h post treatment. Our 16S rRNA PCR data showed the enhanced bacterial abundance after the treatment (Figure 2D). We measured the body weight of the treated mice and did not find significant changes. We did not observe any inflammation in the intestine after collecting tissue samples. The compositions of *Salmonella*, *Lactobacillus* and *Bacteroides Fragilis* did not change in the melanized bacteria-treated mice (Figure 2E).

**Figure 3:**
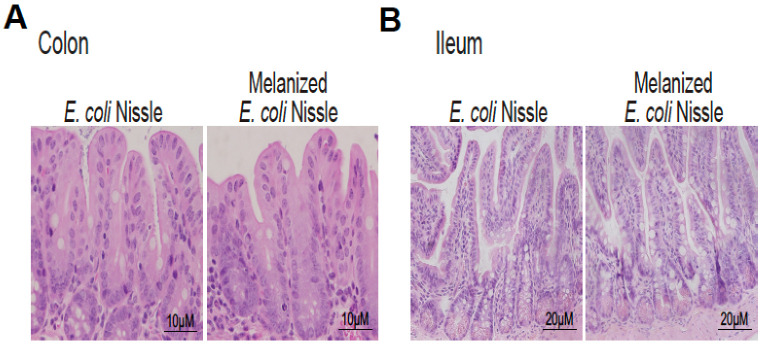
The morphology of colon and ileum did not change in the CD-1 mice one-gavaged with probiotic *E. coli* Nissle or melanized *E. coli* Nissle in H&E staining. (**A**,**B**) Representative H&E images were from a single experiment and were representative of 6 mice per group.

The morphology of colon and ileum did not change in the CD-1 mice one-gavaged with probiotic *E. coli* Nissle or melanized *E. coli* Nissle in H&E staining (Figure 3A,B).

## 4. Discussion

There is a need to develop mitigators of radiation injury for cancer patients undergoing radiation therapy or after mass exposure of population to ionizing radiation. Recently, soluble allomelanin showed promise as a mitigator of radiation injury when administered to irradiated mice up to 48 h post-irradiation [31]. In addition, melanized *E. coli* Nissle has been generated with the purpose of being used as a potential mitigator of radiation injury. Thus, before embarking on the systematic evaluation of soluble melanin and melanized bacteria as radiation countermeasures, it was important to demonstrate the safety of these agents towards microbiome.

It is the first time to report the melanized *E. coli* Nissle used *in vivo*. The melanized *E. coli* Nissle could be detected at 3, 12, and 24 h post treatment. The number of melanized bacteria increased at 3, 12, and 24 h post treatment compared to the before treatment in CFU count and by *E. coli* 16s rRNA RT-PCR. These data showed the colonization ability of the melanized *E. coli* Nissle in less than 3 h and its consistent colonization in the colon over 24 h post one oral gavage. In regard to soluble allomelanin, it proved to be safe to gut microbiome, as it did not cause any body weight change in treated mice and did not change the compositions of *Escherichia coli* and *Lactobacillus* components of microbiome when compared to the untreated mice. Importantly, we did not observe any inflammation in the intestine for both agents. The limitation of our study is that we did not test the general microbiome community and we only observed the mice treated with melanized *E. coli* Nissle. The current study was designed to address the acute effects of melanized *E. coli* Nissle. Survivors of acute radiation injury develop delayed effects of radiation injury. Thus, we will study the long-time effects and provide safety information of mice with melanized *E. coli* Nissle over months. Such studies are planned for the future.

In conclusion, our results demonstrate the safety of soluble melanin and melanin-producing bacteria and will support the future studies to treat radiation-induced injuries and restore dysbiosis.

## Figures and Tables

**Table 1:** Real-time PCR Primers.

Primers Name	Sequence
*E.Coli* F	5′-CCTACGGGAGGCAGCAGT-3′
*E.Coli* R	5′-CGTTTACGGCGTGGACTAC-3′
*Salmonella* F	5′-CACAAATCCATCTCTGGA-3′
*Salmonella* R	5′-TGTTGTGGTTAATAACCGCA-3′
*Lactobacillus* F	5′-AGCAGTAGGGAATCTTCCA-3′
*Lactobacillus* R	5′-CACCGCTACACATGGAG-3′
*Bacteroides fragilis* F	5′-CTGAACCAGCCAAGTAGCG-3′
*Bacteroides fragilis* R	5′-CCGCAAACTTTCACAACTGACTTA-3′
Universal bacteria F	5′-TCCTACGGGAGGCAGCAGT-3′
Universal bacteria R	5′-GGACTACCAGGGTATCTAATCCTGTT-3′

## Data Availability

Not applicable.
